# From jurisdiction to veridiction: Expertise and economization of immunization policy in the United States

**DOI:** 10.1080/03085147.2025.2517951

**Published:** 2025-06-26

**Authors:** Dmitrii M. Zhikharevich, Katharina T. Paul

**Affiliations:** a Dmitrii M. Zhikharevich Department of Political Science, University of Vienna, Kolingasse 14-16, 1090 Vienna, Austria. E-mail: dmitrii.zhikharevich@univie.ac.at; b Katharina T. Paul (corresponding author), Department of Political Science, University of Vienna, Kolingasse 14-16, 1090 Vienna, Austria. E-mail: katharina.t.paul@univie.ac.at

**Keywords:** vaccination, economization, public health, cost–benefit analysis, expertise, Foucault

## Abstract

Economic valuations play an increasingly important role in contemporary vaccination governance. Focusing on the United States, this paper presents a genealogy of economization in immunization policy. Borrowing from Foucault, it argues that economization occurs in two distinct modalities: juridical and veridical. In the former case, economization serves the purpose of ‘doing justice’ to an issue or practice. In the latter case, it asks what must be true about the world for an issue or practice to be justified. Drawing on published, archival and oral history sources, we show how immunizations in the United States first became economized in a juridical modality in the 1960s. We then trace their veridical turn in the 1970s by looking at the traffic of ideas and individuals between the Centers for Disease Control and the Harvard School of Public Health.

## Introduction

Contemporary vaccine governance is saturated by increasingly standardized economic evaluations, taking the form of cost–benefit (CBA) or cost-effectiveness analysis (CEA).^1^ Economic metrics are integral to the functioning of ‘philanthrocapitalism’, providing its actors with epidemiological accountability (Reubi, 2018) and charismatic authority by tracing the impact of their ‘mega-giving’ (McGoey & Thiel, 2018). Vaccination campaigns also require various ‘evidential practices’ (Ehrenstein & Neyland, 2018), ranging from randomized control trials (RCTs) with the strongest ‘evidentiary charisma’ (Kelly, 2018), to the less visible ones like modelling and impact assessment. In these practices, problems are defined, solutions devised, and resources get mobilized. This paper traces the emergence of one such practice, insufficiently explored in the literature: the economization of immunization.

Historical work suggests that economization of immunization predates the increasing privatization of vaccine production and governance, associated with the rise of global health in the 1990s. Thomas (2022) argues that immunizations first became economized when the World Health Organisation (WHO) Expanded Programme on Immunization turned to CBAs in the 1970s to navigate its financial and political challenges by appealing to ‘trust in numbers’ (Porter, 1993). In this paper, we present a complementary genealogy of economization of immunization, rooted in the North American context, and the two institutions exerting crucial influence on health policy globally: the Centers for Disease Control and Prevention (CDC) and the Harvard School of Public Health (HSPH). The United States has been one of the major developmental hubs of health economics (Moos, 2015) and, as we demonstrate below, of early applications of economic thinking to immunizations. Unlike elsewhere, such as the United Kingdom and Sweden, these early applications were realized not by economists, but by public health and policy experts, many of them affiliated with the CDC and/or HSPH. Thus, the US case is particularly instructive for understanding how elements of economic expertise travel beyond the discipline of economics and into the realm of policymaking (Hirschman & Berman, 2014). Examining the institutional sedimentation of expertise is especially relevant now, as the new US administration threatens to disrupt policy continuity in public health.

This paper uncovers a longer thread of economization of immunization, stretching back to the 1960s and the ascendancy of the economic style of reasoning in American public bureaucracies (Berman, 2022). The first self-conscious applications of economic analysis to immunizations, in the form of CBAs, occurred at the CDC in this context. Aiming to justify immunization programmes targeting ‘mild’ childhood diseases (Conis, 2014), they quantified the economic value of immunizations by representing it as a share of national expenditure. In the latter part of the 1970s, a new problematization of healthcare in America took shape, focusing on the rising healthcare costs, and linking them up with the excessive use of medical technology. In response to this new problematization, scholars at the Harvard’s Center for Analysis of Health Practices (CAHP) positioned CBAs-CEAs as tools for rationalization of allocative decisions in health and medicine. In what follows, we trace how this new form of economic analysis was applied to immunizations. We argue that the modality of economization of immunizations has changed from ‘jurisdiction’ to ‘veridiction’ (Foucault, 2009 [1979]) and thus point to the ambivalence of economization (Griffen & Panofsky, 2021).

We adopted a genealogical mode of analysis in the broadly Foucauldian idiom (Foucault, 2009 [1979]; Mennicken & Miller, 2014). Our exploration of the professional literature on vaccine policy (Miller & Hinman, 2013), Web of Science (WoS) database, and historical bibliographies indicated the importance of CBA-CEA.^2^ We took the earliest CBA of immunization (Axnick *et al.*, 1969) as a starting point for our analysis, and identified other turning points in the history of economic analyses of immunizations. Finally, archival and oral history sources enabled a focused reconstruction of the processes of economization.

## Economization between jurisdiction and veridiction

Recent literature on economization largely follows Çalışkan and Callon (2009, p. 370), exploring how ‘behaviours, organizations, institutions and, more generally, objects’ are constituted as economic in various settings. Here, scholars point to styles of reasoning (Berman, 2022) and cognitive infrastructures (Hirschman & Berman, 2014) that interact in ambivalent ways. Thus, economists’ struggle for recognition within their professional field can condition whether economic policy devices will be appropriated as devices for ‘seeing’ or for ‘choosing’ (Griffen & Panofsky, 2021; Hirschman & Berman, 2014). Moreover, to be consequential, economization does not have to directly influence policy decisions: calculative tools like CEA can be used as technologies of economic justification for existing policies (Griffen & Timmermans, 2020), while the economic style of reasoning can inform the way policy problems are framed, rather than dictating specific solutions (Berman, 2022). These arguments resonate with the accounting literature that emphasizes the contingency of the links between calculative technologies that help constitute the ‘economic’ domain, and the ‘historically varying ideas or rationalities’ of government that ‘require and inspire’ them (Mennicken & Miller, 2014, p. 18). In a different theoretical idiom, economic valuation is said to operate within a set of assumptions about its purposes and limits, embedded in the cultural and institutional contexts in calculations are deployed (Fourcade, 2011). In short, calculative technologies derive their meaning and purpose from the projects of governing they help to operationalize (Mennicken & Miller, 2014, pp. 19–20).

Building on this work, we explore the ambivalence of economization by engaging with Foucault’s (2009 [1979]) distinction between ‘jurisdiction’ and ‘veridiction’. In the lectures on biopolitics, Foucault reconstructs how the practice of government was embedded into a particular ‘regime of truth’ of political economy. As a result, the market ceased to be ‘a site of justice’ and became reconstituted as ‘a site of veridiction’, ‘of verification-falsification for governmental practice’ (Foucault, 2009 [1979], pp. 31–32).

[W]hat is discovered at this moment, at once in governmental practice and in reflection on this governmental practice, is that inasmuch as prices are determined in accordance with the natural mechanisms of the market, they constitute a standard of truth which enables us to discern which governmental practices are correct and which are erroneous. […] it is the natural mechanism of the market and the formation of a natural price that enables us to falsify and verify governmental practice when, on the basis of these elements, we examine what government does, the measures it takes, and the rules it imposes […] the market determines that good government is no longer simply government that functions according to justice … no longer quite simply one that is just. The market now means that to be good government, government has to function according to truth. (Foucault, 2009 [1979], p. 32)

Foucault (2009 [1979], p. 34) discerned a similar pattern in the history of disciplinary institutions: e.g. the shift from a jurisdictional question (‘what have you done?’) to the veridictional (‘who are you?’) in the penal practice. This distinction pertains to economization, too: On the one hand, economization enables access to a repertoire of justification, with its tools mobilized to quite literally ‘do justice’ to an issue by making visible its economic worth or impact (cf. Fourcade, 2011). Modelling the cost and (societal) value of pandemic measures, including vaccination during the COVID-19 pandemic, or economic quantification of the value of gender equality to the global economy (e.g. IMF, 2024) are cases in point. On the other hand, economization can lead to the constitution of ‘a standard of truth … to discern which governmental practices are correct’ (Foucault, 2009 [1979], p. 32), and its tools mobilized to reveal the said truth, as technologies of veridiction. The plethora of evaluations of pandemic measures, such as the UK Public Inquiry, staged as a veridical testimony, are instances of such practices. We suggest that, while economization often begins in a juridical modality, responding to the need to justify existing practice in the face of institutional or environmental pressures (Thomas, 2022), it can shift to a veridical modality, making possible new truth claims about the economized practice and turning them into evaluation standards for it. Such shifts depend on the problematizations (Miller & Rose, 2009) that exist in the practical domains being economized, and on the actors’ ability to translate their local concerns into the language of economics.

In what follows, we reconstruct the shift from jurisdiction to veridiction in the economization of vaccinations in the United States in two broad episodes. The first episode occurred in the 1960s, when immunizations at the CDC were economized under a ‘jurisdictional’ modality, to ‘do justice’ to vaccination programmes by rendering their public health value more visible, responding to the public health problem of ‘mild diseases’. The second episode occurred in the middle of the next decade, marking the shift towards veridiction. Instead of asking whether a particular policy decision was justified economically (assuming its public health value), economic evaluations now asked (explicitly or not) what must be true about the world for a decision to make economic sense.

## Economic style of reasoning and the problem of ‘mild’ diseases at the CDC

Our analysis of the literature points to the CDC in the 1960s as the source of the first self-conscious economic evaluations of immunizations. They assumed the form of CBA and were inspired by the health-economic framework of ‘investing in people’. Thus, to better understand the economization of vaccines, we first need to attend to health economics.

Health economics emerged in the United States in the 1950s and consolidated as a subfield in the early 1960s, in parallel with the economics of education (Fein, 1971; Klarman, 1979; Moos, 2015). Both fields were concerned with ‘human resources’ and deployed the concept of human capital to capture the contribution of ‘the changes in the quality of people to economic growth’ (Fein, 1971; Mushkin, 1962, p. 129). Early health economists conceptualized health as a kind of human capital, and healthcare as an ‘industry’ within the national economy, focusing on the efficiency of its organization and the use of its resources (Mushkin, 1958, p. 790). In this framework, public health was conceived of as a public good whose provision depended on government investment. But, as a form of human capital, it also promised a higher return on a dollar ‘invested in people’ (Klarman, 1965; Mushkin, 1958; Weisbrod, 1961). The development of national-scale health statistics, exemplified by the creation in 1960 of the National Center for Health Statistics, enabled assessments of the economic impact of disease (Rice, 1981). By the middle of the decade, the methodology for the ‘cost-of-illness’ studies (Rice, 1967) became established: a disease’s impact could be quantified as a combination of its direct (medical) and indirect (lost productivity) costs, understood as claims upon the ‘Nation’s resources’, expressed in monetary terms (Rice, 1967, p. 424). Put into a relationship to the statistical whole of the ‘American economy’, these costs were thus *economized* (cf. Berman, 2014).

The ambitious social welfare programmes of the 1960s, including Medicare and Medicaid, created a demand for economic expertise in the federal government (Berman, 2022). As a result, the academic concern with human capital was elaborated into a programme of governing public health as a range of competing ‘investments in people’ (Miller & Rose, 2009). Rather than showing ‘that education had a high yield and that disease was costly’, economists were now asked ‘what specific programmes could – in the real world – lower the incidence of disease and by how much’ (Fein, 1971, pp. 193, 195; Mushkin, 1962). Under this framing, the benefits of a health programme were equivalent to the economic costs of the illness it helped to avoid (Klarman, 1965; Rice, 1967) and could be treated as returns on the investment of national resources. In this context, CBAs, initially codified for the analysis of public infrastructural projects in the 1930s (Porter, 1993), emerged as a technology to operationalize the policy idea of ‘investing in people’ (Dorfman, 1965), with economists in charge of ‘identify[ing] an objective function for decisions on expenditures’ (Mushkin, 1962, p. 153). These developments became particularly prominent during Lyndon Johnson’s presidency, when the federal government embraced the specific variety of the economic style of reasoning developed by the RAND Corporation (Berman, 2022). Animated by the aspiration of rationalizing government decision-making, RAND economists developed an analytical approach to the ‘problems of choice’, known as systems analysis. Its public policy avatar, the Planning Programming Budgeting System (PPBS), sought to tie together federal agencies’ goal setting and budgeting via cost-effectiveness criteria (Berman, 2022; Knafo *et al.*, 2019). PPBS failed to rationalize government decision-making but made an ‘enormous indirect impact’ by embedding the economic way of thinking about policy within federal agencies, as they rushed to establish policy planning and evaluation offices, the ‘beachheads of economic reasoning’, emphasizing choice among alternatives, cost-effectiveness, and quantitative analysis (Berman, 2022, p. 58).

The economization of immunization programmes also started in this context. Initially, health economists treated vaccination and disease control as an illustration of the need for government investment into public goods whose social value far exceeded its private marginal value to individuals (Mushkin, 1958, p. 790; Weisbrod, 1961, p. 27; Fuchs, 1972, p. 224). In the 1960s and the early 1970s, empirical economic analyses of vaccines were relatively rare, ranging from hypothetical modelling exercises to measurements of vaccine benefits taken as returns on investments in vaccine research (Weisbrod, 1961, 1971). The application of the economic framework of ‘investing in people’ operationalized through CBA techniques took place in a policy, rather than an academic environment: the Communicable Disease Center (CDC) of the US Public Health Service. Established in 1946, the CDC gained credibility in the 1950s for its efficient handling of the outbreaks of polio and Asian influenza and continued to expand its efforts in the 1960s (Etheridge, 1992). In 1967, the CDC became the National Communicable Disease Center within the Department of Health, Education and Welfare, and created its own Office of Planning and Analysis, later renamed the Office of Program Planning and Evaluation (OPPE) (CDC, 1968). Like other agencies unable to afford an ex-RAND economist to run the new office, the CDC adopted the next best option, hiring people with some training in economics, for whom the RAND-style approach would seem intuitive even without prior exposure (Berman, 2022, p. 56). The OPPE led the agency’s early experiments with CBAs of epidemics and immunizations, including an influential study of measles immunization (Axnick *et al.*, 1969), that became a ‘classic of its kind’ (Russell, 1986, p. 31, note 89) and thus deserves to be examined in some detail.

## Benefits of measles immunization and juridical economization

Co-authored by two economists and one epidemiologist, the study aimed ‘to quantify the national impact’ of measles immunization since the licensure of the vaccine in 1963 (Axnick *et al.*, 1969). The authors defined the net immunization benefit as the difference between the ‘economic costs’ of measles with and without immunization, less the cost of immunization programme itself. Drawing on the conventions of cost-of-illness studies, the paper quantified the impact of measles using the CDC’s own data, as well as economically, as medical expenses and the value of productivity lost from work absenteeism in adults and mental retardation in children, discounted at 4 per cent. Following the same conventions, ‘important but difficult-to-measure costs such as misery and unhappiness’ were excluded. The study’s central point was the sharp decline of the incidence of measles following immunization: from four million cases in 1963 to 250,000 in 1968, with a correspondent dramatic reduction of costs. In addition to saving the nation 1.6 million workdays and 291,000 additional years of ‘normal and productive life’ for 4,000 persons (Axnick *et al.*, 1969, p. 677), five years of measles immunization created an economic benefit of $531.5 million, with $423 million being savings (Axnick *et al.*, 1969, p. 679).

The measles study became a flagship product of the CDC, presented at major international conferences and featured in the CDC’s measles surveillance reports (CDC, 1973; Sencer & Axnick, 1973, 1975). The paper elaborated upon the investment-based justification of prevention in addition to the ‘humanistic’ one, a familiar combination in the Anglophone public health since the 1860s (Annual Meeting, 1878; The cost of an epidemic, 1868). But rather than simply *counting* the costs of measles, the CDC study also *economized* them by linking them to a range of statistical inscriptions such as the average earnings, consumer prices, and physician fees aggregated at the level of the national economy (Axnick *et al.*, 1969). Moreover, economization included the *benefits* of immunization, going beyond the non-economic appraisal of vaccination, and was forward-looking, pointing to a positive externality: over time, economic benefits increased with the share of the immune population (Axnick *et al.*, 1969, p. 678).

For the CDC, embracing ‘investments in people’ and CBAs (Sencer & Axnick, 1975, p. 123) offered a way of addressing their concern with the so-called ‘mild’ infectious diseases. After the successful campaigns against polio and smallpox in the 1950s and developments of new vaccines against measles, rubella and mumps in the 1960s, US public health officers reconceptualized these latter diseases as ‘less severe than – and therefore categorically different from – polio, smallpox, and diphtheria, the previous targets of mass vaccination’ (Conis, 2014, pp. 6–7). The presence of these ‘mild’ diseases distinguished the United States from the developing world and necessitated an additional justification for immunizing against them (Cockburn, 1973; Sencer & Axnick, 1973; Axnick & Lane, n.d.). Economization offered a way of problematizing ‘mild’ diseases from a new perspective. CBAs thus became a *technology of justification* (Griffen & Timmermans, 2020) of the costs of ‘mild’ disease immunization that did not compromise their ‘humanistic benefits’ and conferred on them some of the cultural prestige of quantification (Porter, 1993):

Possibly the most important aspect of these statistics [sic!] is that they make explicit the full magnitude of the harm that can be done by a ‘mild’ children’s disease. (Axnick *et al.*, 1969, pp. 678–679)

The humanistic benefits resulting from prevention … are obvious. Less obvious, however, are … economic benefits, translated into dollars, that the administrator of a health service can use to justify the seemingly high cost of vaccination programs and to raise the priority for the funding of vaccine-preventable disease programs. The expression in economic terms of the savings accrued by preventing disease requires a new parlance for physicians … The benefits derived from immunization must be described in terms that can be added or subtracted. (Witte & Axnick, 1975, p. 205)

Speaking at an international symposium on vaccination in 1973, the CDC director David Sencer called CBAs ‘tools that the administrator of a health service can use to justify what may seem to be inordinate cost’ (Sencer & Axnick, 1973, p. 37). This view was endorsed by the chief medical officer of the WHO Viral Diseases Unit, describing CBAs as way ‘to persuade the economic and health planners to provide the funds … for continuing immunization after a disease has been brought under control’ (Cockburn, 1973, p. 9).

In their comments on the ‘cost–benefit thinking’ in public health (General panel discussion, 1975; Sencer & Axnick, 1975), the CDC experts tended to preserve the distinction between the ‘humanistic’ and the economic benefits of immunizations, the latter serving to make the former better visible. This was also reflected in the stated ambitions of the ‘economic-type of thinking’: to quantify and inform, to offer an additional perspective or dimension (Sencer & Axnick, 1975, p. 123; Schwab, 1968; Shavell, 1969), ‘to help an administrator identify and evaluate the various ramifications of the options open to him’ (Axnick *et al.*, 1969, p. 679). Economization thus offered a way of capturing the ‘trust in numbers’ (Porter, 1993), as well as amplifying the otherwise invisible value of preventing ‘mild’ diseases, reinforcing, rather than challenging, the ‘humanistic’ justification of immunization accepted in public health. In our terminology, economization here was juridical, with CBAs serving as technologies of economic justification (Griffen & Timmermans, 2020), mobilized to make an economic case in support of public health decisions, such as the termination of smallpox vaccination (Axnick & Lane, n.d.) or the introduction of measles immunization (Axnick *et al.*, 1969). In 1980, the Congressional Office of Technology Assessment (OTA) published a report on the uses of CBA-CEA in federal agencies, quoting an ex-CDC staffer saying: ‘we used CBA to evaluate tuberculosis vaccination programs, and I can tell you that even then, the answer came before the analysis was ever done, and it always justified the decision’ (OTA, 1980a, p. 139). Despite the references to decision-making, the justification of existing prevention activities ‘where new scientific evidence or public concerns threatened their existence’ remained the primary use of economic evaluations of immunizations through the 1970s (Corso *et al.*, 2002, p. S11).

## Harvard seminar on health and medicine and the problem of medical commons

While CBA and CEA became established technologies of economic justification (Griffen & Timmermans, 2020) for immunization programmes, the notion that healthcare expenditure was an investment in human capital promising unquestionable, if perhaps not immediately obvious, benefits (as in the case of ‘mild’ disease immunizations), was increasingly considered too optimistic. Between 1965 and 1973, the share of health expenditures in the federal budget increased from 4.4 per cent to 11.3 per cent, raising the issue of cost control (Henig, 1997, p. 184). For economists, this spurred an interest in the role of medical technology as a potential driver of healthcare costs (Altman & Blendon, 1979; Wagner, 1979). In 1975, a health programme was created at the OTA; staffed by economists, it embarked on an ambitious project of evaluating medical technologies using CBA and CEA, including vaccines (OTA, 1976, p. 4). The changing rationale for the use of CBA-CEAs emphasized the assumption of resource scarcity, making their structure conform ‘with an attitude … growing in health policy … that resources are limited … choices must be made among alternative technologies and programs, and … it is preferable to realize the greatest health benefit per dollar’ (Willems, 1979, p. 47).

The new problematization of medical technology implied a more pessimistic view of externalities in health, articulated, among others, by Howard Hiatt, an influential medical scientist, physician and health administrator. In 1975, Hiatt, then dean of the HSPH, described the problem in terms of Garrett Hardin’s analysis of the ‘tragedy of commons’ (Hardin, 1968; Hiatt, 1975). Medical practices that pitched individuals against society – expensive, technology-intensive therapies, interventions of unproven effectiveness, and treatments of preventable conditions – drained the medical commons. To address this, Hiatt (1975) advocated a thorough re-evaluation of healthcare, tying together the problem of rising costs and quality of medical decision-making, and proposing technology assessment as a solution. In effect, he articulated another programme for governing healthcare, with CBAs-CEAs again serving to operationalize it.

This vision was closely related to Hiatt’s activities as the dean. Appointed in 1972, he found HSPH ‘lacking in almost every respect’ (Rosenberg, 2018, p. 70). One of the oldest institutions of its kind in the country (Henig, 1997), it was a traditional public health school preparing students to run state and local health departments. It was largely isolated from Harvard’s other departments and from ongoing developments in the economic and policy sciences. Breaking this insularity became one of Hiatt’s priorities (Rosenberg, 2018, pp. 62–63), manifested, among other things, by the creation of the CAHP in 1972. As a result, Harvard became one of the first public health schools to include faculty from social and policy sciences (Henig, 1997, p. 11; Rosenberg, 2018, pp. 67–69).

The new Center started off as a regular Seminar in Health and Medicine, co-organized with the Department of Statistics, bringing together more than a 100 participants from the Harvard community and wider Boston area ‘to investigate medical questions by using principles of economics, statistics, law, management, engineering, and biology’ (Henig, 1997, p. 184; Rosenberg, 2018, pp. 69–74). To organize it, Hiatt relied on a list of advisors featuring key representatives of post-war decision sciences (Henig, 1997; Rosenberg, 2018, p. 69).^3^ The Seminar enabled cross-disciplinary work, some of which would later become standard in the field, including the codification of CBAs-CEAs methodology (Shepard & Thompson, 1979; Weinstein & Stason, 1977a). More generally, the Seminar catalysed the application of quantitative expertise to the study of medical decision-making and public health policy. For the HSPH, the Seminar and the CAHP were what the new policy planning offices were for the federal agencies: a ‘beachhead for economic reasoning’, and a training ground for a new, ‘RAND lite’, cadre of public health analysts comfortable with thinking like an economist (Berman, 2022, pp. 58, 65).

The first three years of the Seminar’s work produced two landmark publications responding to the problematization of rising medical costs: a study of hypertension as a public policy problem led by a cardiologist and a policy analyst (Weinstein & Stason, 1976), and an edited volume *Costs, risks, and benefits of surgery* (Bunker *et al.*, 1977), featuring contributions from junior academics participating in the increased cross-pollination between health and policy sciences. Focusing on health problems of exemplary importance and complexity regarding the trade-offs involved, these studies advocated quantitative analysis as a solution to the problem of medical commons articulated by Hiatt. From health planners to physicians, health-related decisions were seen as diffuse and taken without awareness of their consequences for the medical commons; fraught with uncertainty regarding the efficacy of medical practices and technologies; and faced with implicit value trade-offs, with physicians’ willingness ‘to provide everything possible for the patients regardless of cost’ clashing with the societal objective’ of maximizing health benefits per dollar (Weinstein & Stason, 1977b, p. 29).

The solution was to be found in CBA-CEA, combined with decision analysis (DA) to take into account ‘the uncertainty about both the effects and costs of alternative actions and by including valued outcomes such as quality of life’, thus allowing for more flexibility in the formulation of assumptions and conclusions (Pliskin & Taylor, 1977, pp. 11–20; Weinstein & Stason, 1976, pp. 14–15). DA provided a framework for explicit consideration of alternatives by separating outcomes occurring by chance and by choice and specifying the probability of each separately from some measure of its worth (e.g. its effectiveness and costs) (McNeil & Pauker, 1984; Pliskin & Taylor, 1977). Positioned as a rational way of decision-making that recognizes the underlying truth of scarcity (Weinstein & Stason, 1976, p. xi), this approach mandated the use of sensitivity analyses to explore various scenarios and assumed a self-consciously ‘veridical’ stance. Firstly, the task was no longer justification of a presumably ‘good’ policy; to be ‘good’, a policy ‘had to function in according to truth’ (Foucault, 2009 [1979], p. 32) of conflicting interests, value-tradeoffs and uncertain efficacy, revealed by the analysis. Second, it was argued that economic analysis could not ‘make judgments for society, but it can frequently clarify the issue by identifying advantages and disadvantages, quantifying effects, and measuring the resources involved’ (Pliskin & Taylor, 1977, p. 6), explicating the beliefs and values that underlie allocation decisions (Weinstein & Stason, 1976, p. 14). Third, providing such information to physicians and other decision-makers was seen as a possible way ‘to more cost-effective uses of medical resources’ (Weinstein, 1979, p. 66).

In the late 1970s, CAHP scholars developed methodological guidelines and tutorials for CBA-CEA, integrating them with decision analysis and strengthening the veridical view of economic evaluations (Shepard & Thompson, 1979; Weinstein, 1979; Weinstein & Stason, 1977a). Under Hiatt’s leadership, the HSPH emerged as a major hub for the development and application of CBA and CEA in health and medicine, first in research at the CAHP, and later also in teaching. Nevertheless, while vaccines were theoretically included into the broad category of ‘medical technology’ (Hiatt, 1975; OTA, 1976), the developments at Harvard remained largely disconnected from the CDC and immunization policymaking. Having laid out the consolidation of health economics, its early applications to immunizations at the CDC, and the formation of a quantitative approach to health policy at the CAHP, in the next section, we trace how they became entangled with each other.

## From jurisdiction to veridiction: *The swine-influenza decision*

The new style of economic analysis of immunizations was gradually taking shape in the spaces between academia and policy, traversed by individuals who bridged both worlds. One of the key contributors to the veridical turn, Stephen Schoenbaum, spent two years at the CDC’s Epidemic Intelligence Service (EIS) after obtaining his MD from Harvard in 1966. With the CDC’s rather compartmentalized organization in the late 1960s, he had no exposure to the economic analyses conducted by the OPPE but developed an interest in the policy issues surrounding the ‘milder’ infectious diseases like rubella. Taking this interest back to Boston, in 1973, Schoenbaum applied to the HSPH to learn more about policy. The MPH curriculum remained largely traditional, and Schoenbaum’s only encounter with economics there was through an introductory course taught part-time by Ralph Berry, a member of the pioneering cohort of Harvard health economists (Klarman, 1979, p. 375). As part of the coursework, Schoenbaum convinced three classmates to write a paper on rubella vaccination policy, later published in the *New England Journal of Medicine* (Schoenbaum *et al.*, 1976a). The paper compared the costs and benefits of vaccinating 2- and 6-years-old children of both sexes, and 12-years-old girls under 100 per cent and 80 per cent compliance scenarios, finding that the latter strategy would yield greater economic benefits. Moreover, the authors concluded that existing rubella policy was not delivering on either of its possible goals:

[P]olicies aimed at prevention of a maximum number of babies with congenital rubella syndrome, or at maximizing the net economic benefits, or at maximizing the economic benefits per dollar spent would all have led to a choice of policy different from that actually chosen by the United States. (Schoenbaum *et al.*, 1976a, p. 310)

Written as coursework in introductory economics, effectively as an exercise in the economic style of reasoning (Berman, 2022), the paper scrutinized existing vaccination policy against a range of possible alternatives and used CBA to compare them. Rather than simply proclaiming that cost–benefit information ‘is of value to the administrator who must make decisions’ on public spending (Witte & Axnick, 1975, p. 207), it modelled the decision situation as a choice among alternatives and led to a counter-intuitive result (Willems, 1979, p. 48).

This approach was further developed in the study of the swine-influenza vaccination (Schoenbaum *et al.*, 1976b). In early 1976, atypical influenza A related to the 1918 pandemic was discovered at Ford Dix, New Jersey. Following the CDC’s advice, President Ford announced a mass vaccination campaign, which was implemented swiftly, but was terminated prematurely due to the occurrence of severe side effects and the lack of a pandemic. As a result, vaccination and its governance were brought to the ‘public trial’ (Karzon, 1977; Neustadt & Fineberg, 1978).^4^ With his history of interest in immunization policy, Schoenbaum saw an opportunity for an expert intervention and formed a collaboration with two like-minded colleagues who belonged to the same social and intellectual circle. The first co-author, Barbara McNeil, was Schoenbaum’s classmate from Harvard Medical School; in 1973–1976, she participated in Hiatt’s seminar on health and medicine. The second co-author, Joel Kavet, had an economics degree and was writing a doctoral dissertation at the HSPH on influenza control policy when he met Schoenbaum at EIS. Building on the cost–benefit model developed at the CDC by Norman Axnick, head of the OPPE and a co-author of the measles study (Axnick *et al.*, 1969), in his 1972 dissertation, Kavet provided an economic analysis of the federal influenza control policy, endorsing it as ‘justifiable’ on economic grounds (Kavet, 1972). Schoenbaum knew that Kavet had an ‘economic model’ of influenza control and that McNeil was interested in decision analysis: ‘it struck me that we needed to put these things together’.^5^ The three co-authors would meet at Schoenbaum’s house for dinner and work on the analysis. In addition to bringing together the economic analysis of vaccinations (Kavet) and the new quantitative methods developed at Harvard (McNeil), the paper used the Delphi technique, virtually unknown in public health literature at the time.^6^

The study departed from previous analyses of immunizations in three important respects. Like earlier CDC studies, the paper analysed a policy decision that had already been made. However, reversing the logic of these studies, the authors attempted to reconstruct the assumptions that *should have been made* by the policymakers, thereby also reconstructing the conditions under which the policy of universal immunization would have been justified: ‘was this reasonable or not to have made this decision?’.^7^ The standard of reasonability was economic: obtaining the maximum net benefits and making the best use of limited resources. Thus, rather than assuming the economic value of the programme in addition to its ‘humanistic benefits’, this assumed value was called into question – and found wanting, since the expected net benefits were not made maximum under a universal immunization scenario (Schoenbaum *et al.*, 1976b, p. 764).

Second, economic calculations were performed under different assumptions about the probability and attack rate of the epidemic, using the estimates obtained via the Delphi survey of epidemiologists, and subjected to extensive sensitivity analyses. Armed with these tools, co-authors explicitly took issue with the way vaccination policy was determined: instead of relying on an opinion of a group of experts briefed about the situation, they suggested to elicit estimates for the unknown variables from the experts and conduct a formal analysis to formulate policy (Schoenbaum *et al.*, 1976b, p. 763). Rather than using CBA to provide an ‘additional perspective’ for the policymakers without challenging their judgment, the co-authors endorsed formal analysis which transformed qualified expert judgment into a mere ‘input’ (cf. Knafo *et al.*, 2019; Porter, 1993). In the discussion that ensued after the publication, they further emphasized the usefulness of the formal approach: ‘ultimately … a decision must be made’, yet if ‘the probability of disease is low and … there is no model for a guide to decision making, intelligent persons frequently differ on the “conservative” approach – to vaccinate and incur reactions, or not to vaccinate and risk disease’ (Schoenbaum *et al*., 1977, p. 47).

Third, in addition to calculating the costs of epidemic in the absence of preventive effort ($6 billion for the general population and $3 billion for the high-risk groups), the paper demonstrated that the immunization net benefits would vary depending on the target group, the costs of vaccine administration, and the rate of its acceptance. Universal immunization yielded maximum benefits only under a set of strict assumptions: the 10 per cent probability of an epidemic and the costs of administering vaccine below $0.50 per person. Benefits would exceed the costs with the acceptance rate of at least 59 per cent, if the vaccine was restricted to people older than 25. Kavet’s (1972) earlier work also compared CBAs of vaccination against different strains of influenza but otherwise remained broadly in line with the CDC approach. By combining elements of CBA and formal decision analysis, the swine-influenza paper elevated economic analysis to much more than a tool of quantification: it offered a framework in which different substantive assumptions, or decision ‘inputs’, could be made explicit, explored and compared, as well as distinguished from the confounding influences of politics:

[O]nce it [a formal analysis] has been completed, the framework can be used for analysing alternative strategies. The formal analysis indicates whether a particular strategy will break even or yield net benefits, and *it can identify the conditions for obtaining maximum net benefits*. In the absence of comparable benefit–cost data for other public-health programs, the final decision to expend limited resources on influenza vaccination rather than on some other program incorporates political and social considerations. (Schoenbaum *et al.*, 1976b, p. 763, emphasis added)

The new style of economic analysis of vaccinations differed from the early studies conducted by the CDC in tone and emphasis. Adopting Foucault’s (2009 [1979]) terms, this difference can be described as one between ‘jurisdiction’ and ‘veridiction’. Instead of justifying an immunization programme economically, it asked what must be true of the world for this programme to be justifiable, necessitating a veridical exploration within the limits determined by the economic criteria of CBA. The analysis was no longer about ‘doing justice’ to the programme whose value was assumed, but subjected this assumed value to critical scrutiny, making it contingent on the validity of substantive assumptions about the relevant aspects of the world, the disease, and the vaccine. From a technology of economic justification (Griffen & Timmermans, 2020), CBA thus became a technology of veridiction: of ‘verification-falsification’ of immunization policy (Foucault, 2009 [1979], pp. 32-33).

## From costs to decisions and the Harvard-CDC connection

The swine-influenza paper established a bridge between the economic analyses of immunizations and the new developments in the quantitative health policy analyses taking place at Harvard, making them mutually translatable. Soon after the publication, it was included in reviews and bibliographies of the emerging methods of CBA-CEA and their applications to vaccinations (OTA, 1979; Willems, 1979; Willems & Sanders, 1981). More importantly, it resonated with public health practitioners who were anxiously following the swine-flu vaccination campaign and its outcomes, spurring some debate on the merits of formal analysis. According to a former head of the CDC’s Immunization Division, the paper destabilized some intuitively accepted assumptions about vaccine policy decision-making by using decision analysis to show that universal immunization was not necessarily the best course of action.^8^

Nevertheless, the new style of policy analysis remained largely disconnected from policy making. At the CDC, the OPPE staff remained interested ‘in analysing what do we do and why do we do it, and how could we justify it beyond the science of the individual units of CDC’ (Koplan, 2016). Here, too, ideas travelled via networks built by people. The career of Jeffrey Koplan provides a particularly instructive illustration. A medical doctor by training, Koplan began his career at the CDC’s EIS, worked in the Smallpox Eradication Program and in the CDC missions abroad. In 1977, the CDC sent him to the HSPH for career development. At Harvard, Koplan became ‘interested in … how one can quantitatively approach decision-making’ (Koplan, 2016). This approach became central to his study project, an analysis of the risks, benefits and costs of pertussis vaccination to see if it would support the existing policy (Koplan & Hinman, 1987, p. 71), with Schoenbaum, then at Peter Bent Brigham, and Milton Weinstein, one of the leading scholars at CAHP, acting as collaborators.^9^ The study responded to the ongoing controversy about vaccine side effects in the United Kingdom that had triggered a sharp decline in vaccine acceptance and put its value into question (Koplan *et al.*, 1979, p. 906). Using a decision tree, it modelled the effects of alternative vaccination strategies on pertussis morbidity, mortality and direct medical cost, under different assumptions about vaccine coverage, complications and efficacy, as well as severity and incidence of the disease. The resulting benefit–cost ratio of 2.6–1 in favour of the vaccination programme was then subjected to sensitivity analyses, concluding that, although under highest estimates of reaction rates the results are less clear-cut, the benefits of vaccination exceeded the risks, and savings exceeded the costs. The authors also pointed to the value of the analysis as a ‘framework for examining the controversy over pertussis vaccine’ (Koplan *et al.*, 1979, p. 910). After receiving his MPH in 1978, Koplan returned to the CDC to join the OPPE, becoming its assistant director in 1981:

I came back with a bee in my bonnet about applying it more broadly at CDC, and in particular economic analysis, using that decision-analytic model. […] And I thought this was an area that CDC really needed to beef up, basically, economic elements of public health programs. So, over the course of my time in that position […] with proselytizing doing these economic analyses, I went trying to make relationships all over CDC […] and we did them, multiple ones, for every immunization. (Koplan, 2016)

Koplan turned OPPE into the agency’s centre for economic evaluations and decision analyses (Corso *et al.*, 2002; Messonier, 2006). During this ‘very fertile and exciting time’, he tried to overcome the insularity of the CDC’s individual units by developing relationships with them, while promoting economic analyses of immunizations and other public health activities (Koplan, 2016). Although some of these studies were conducted ‘largely to justify and quantify … economic values’ of existing vaccines (Koplan, 1988, p. 407; Koplan & Axnick, 1982), he advocated the ‘veridical’ view of economic analysis, stressing the usefulness of a ‘structured framework’ for analysing public health controversies or decisions that must be taken under uncertainty. Structuring the decision process was the primary function, with adding costs to decision analysis ‘a logical next step’ (Koplan, 1988, p. 407). Speaking at the CDC’s Immunization Conference in 1981, he made a point that ‘a final advantage is that the analysis is not set in concrete. As better data are acquired or circumstance change, the analysis can be altered and different conclusions could result’ (Koplan, 1981, p. 13). Koplan further stressed that ‘many of these analyses have been done by public health professionals well known to us – Axnick, Sencer, Schoenbaum, Lane, and Millar … and have strengthened our prevention efforts, particularly our immunization program’ (Koplan, 1981, p. 14).

The combination of CBA-CEA and decision analysis, initially promoted by the CAHP, was gradually gaining traction at the CDC and elsewhere. When the pertussis controversy reached the United States, the CDC updated Koplan’s original study, using ‘economic evidence’ veridically, as a basis for ‘rational scientific inspection of the relative benefits, risk, and costs,’ policy-setting and identification of the needs for better information (Corso *et al.*, 2002, p. 12). Decision analyses with Delphi surveys were applied to evaluate the policy toward atypical measles (Hinman & Koplan, 1982) and the choice of preventive treatment for tuberculosis (Koplan & Farer, 1980), which contributed to the acceptance of DA and CEA ‘as helpful adjuncts when faced with difficult public health decisions’ at the CDC (Koplan, 1988, p. 408). In 1982, these analyses were used to develop the indications for the new hepatitis B vaccine (Mulley *et al.*, 1982), and to calculate the cutoff points where vaccination becomes more economical than screening (CDC, 1982; Koplan, 1988). Three years later, the Institute of Medicine (IOM) suggested a combination of CEA and DA for setting vaccine development priorities in the United States (Institute of Medicine, 1985). ‘Designed as an aid to decision-making and not as a definitive answer to the selection process’ (Institute of Medicine, 1985, p. 2), it prescribed sensitivity analyses to mitigate the problem of uncertainty, decision analysis for the problem of value judgments in ranking the diseases, and the use of a structured framework to accommodate new data, conceding the impossibility of complete information. In 1983, a CDC decision analysis with a Delphi survey, endorsing the continuation of using oral polio vaccine, rather than inactivated one (Hinman *et al.*, 1988), was used by the IOM to reassess polio immunization recommendations (Koplan, 1988, p. 409).

Koplan’s ‘proselytizing’ work at the CDC also continued. From 1979 to 1988, he served as Assistant Director and then Director of the CDC’s Preventive Medicine Residency programme, introducing decision science and health economics into the training, and bringing several CAHP members from Harvard as visiting faculty (CDC Archive, n.d.). In 1992, Koplan and the CDC director William Roper introduced ‘prevention effectiveness’ (Meltzer, 2016, p. 7; Roper & Thacker, 1993), the CDC’s internal euphemism for economics and decision sciences (Messonier, 2006), into the training of EIS officers, the agency’s main source of new cadres and its main dissemination channel of new concepts through national public health infrastructure (Corso *et al.*, 2002, p. S13). In 1995, prevention effectiveness developed into a postdoctoral fellowship programme in health economics, whose first-generation alumni co-authored a guidebook on economic evaluation and decision analysis for the CDC (Haddix *et al.*, 1996; Skelton & Meltzer, 2017). By 2002, the CDC employed 50 economists, decision scientists and other ‘quants’ (Corso *et al.*, 2002; Koplan, 1988), further strengthening its link with the field of economics (Abbott, 2005). While remote and indirect, we consider these organizational developments as consequences of the initial process of economization, beginning some 25 years before from the CDC’s ‘core’ activities: immunizations.

## Conclusion

This paper traced the process of economization of immunization in the United States in two broad episodes. In the 1960s, the CDC’s OPPE embraced the health-economic framework of ‘investing in people’ to account for the value of vaccination against the ‘milder’ diseases and produced the first self-conscious applications of CBAs to immunization programmes, relating their costs and benefits to the national economy. Economists and public health officers at the CDC used CBAs to justify the continuation of successful ongoing immunization programmes, equating their economic value to the costs of epidemics thereby averted, and used this economic perspective to demonstrate the ‘magnitude’ of vaccination benefits. The economic value of vaccinations as public health goods was seen as complementary to the ‘humanistic’ one: economic quantification mattered to governance, since it offered an additional perspective for the decision-makers, visualizing the otherwise largely invisible impact of immunizations against what were often presumed to be ‘mild childhood diseases’ like measles. Nevertheless, economization remained juridical: CBAs were often conducted to justify already-made decisions and were meant to function as technologies of justification (Griffen & Timmermans, 2020).

In the 1970s, concerns over rising healthcare costs led to the co-problematization of medical decisions and technologies. CBAs and CEAs, with the addition of DA, were positioned as solutions to this problem. Harvard’s CAHP, under the leadership of Howard Hiatt, emerged as an important hub for quantitative expertise in modelling health-related decision situations, as well as for the training of a new breed of experts, versed in both medicine or public health and quantitative policy analysis (Berman, 2022). The controversy around the swine-flu vaccination in 1976 attracted critical attention to decision-making processes, providing an opportunity to apply the new expertise to immunization policymaking. Employing such techniques as decision trees and sensitivity analyses to explore various assumptions about the underlying epidemiological processes, the new style of economic evaluations was no longer limited to the task of visualizing the underappreciated benefits of immunization programmes; instead, it presented itself as a structured framework for decision-making, especially suited for complex, value-laden, uncertain situations.

Drawing on Foucault (2009 [1979]), we describe this shift as one from ‘jurisdiction’ to ‘veridiction’: instead of justifying an immunization programme economically, economic evaluations now asked what must be true of the world for this programme to be justifiable. Economization is not unitary and occurs in multiple modalities. While the shift from jurisdiction to veridiction may obtain in other policy domains, the institutional and organizational conditions that enable or forestall such shifts warrant further research. One promising direction would be to explore how modalities of economization interact with organizational processes of decoupling, and whether veridical turns lead to tighter couplings of policy and practice (Bromley & Powell, 2012). On a different scale, the shifts in the modality of economization can be related to different forms of linkage between ecologies of policy and economics (Abbott, 2005; Griffen & Panofsky, 2021). Synthesizing across these literatures would allow for the development of a process model of economization.

In the US case, the appeal of economic reasoning was particularly strong during fateful controversies, or moments of valuation, such as the swine flu and pertussis controversies in the 1970s. Having begun as a form of justification, economization made possible other economic arguments, including that prevention is not necessarily cost-saving (Russell, 1986). More importantly, it helped immunization economics, as a combination of cost-effectiveness and decision analysis, to emerge and influence policy planning and debate. Nowadays, increasingly standardized economic evaluations and evidence critically shape vaccine valuations, from research and development to regulation and policymaking, nationally and globally. With the (re)current problematizations of healthcare expenditure on many political agendas, CBAs and CEAs will likely grow even more significant. Unpacking the historical emergence of this expertise is critical, but future research must address current problematizations of vaccination, too. In particular, as a new cadre of government officials in the current US administration question vaccine effectiveness, a broader alliance bringing together credentialed experts and civil society in support of the value of vaccination – be it economic, social, individual or ethical – will be crucial.

## Notes

1 Both types of analysis involve comparisons of incremental costs and benefits of alternative public health interventions. In CBA, benefits are estimated in monetary terms, whereas in CEA natural units are used (e.g., the number of lives saved per dollar spent).

2 Only English-language, US-focused economic analyses of immunizations were included. For bibliographies, see: OTA 1979; 1980a; 1980b; Drummond, 1981; Warner & Luce, 1982; Elixhauser *et al.*, 1993.

3 E.g., the statistician Frederick Mosteller, who contributed to the seminar and later joined the HSPH faculty, the decision theorist Howard Raiffa, and the economist Thomas Schelling, both from the Kennedy School of Government and earlier affiliated with RAND.

4 This section is based on an interview with Stephen Schoenbaum.

5 Schoenbaum interview.

6 Delphi is a technique for structured collection of expert opinion in iterative rounds of anonymous questionnaires and feedback, until consensus is reached, codified at RAND in the 1960s (see Daye, 2018).

7 Schoenbaum interview. See also McNeil & Pauker, 1984, p. 155.

8 Interview with Alan Hinman. For him, the paper also spurred an interest in quantitative policy analysis and decision analysis.

9 Schoenbaum interview.
